# Forecasting Stock Market Indices Using Integration of Encoder, Decoder, and Attention Mechanism

**DOI:** 10.3390/e27010082

**Published:** 2025-01-17

**Authors:** Tien Thanh Thach

**Affiliations:** Faculty of Mathematics and Statistics, Ton Duc Thang University, Ho Chi Minh City 700000, Vietnam; thachthanhtien@tdtu.edu.vn

**Keywords:** recurrent neural networks, long short-term memory, gated recurrent units, encoder–decoder architecture, attention mechanism, Bayesian optimization

## Abstract

Accurate forecasting of stock market indices is crucial for investors, financial analysts, and policymakers. The integration of encoder and decoder architectures, coupled with an attention mechanism, has emerged as a powerful approach to enhance prediction accuracy. This paper presents a novel framework that leverages these components to capture complex temporal dependencies and patterns within stock price data. The encoder effectively transforms an input sequence into a dense representation, which the decoder then uses to reconstruct future values. The attention mechanism provides an additional layer of sophistication, allowing the model to selectively focus on relevant parts of the input sequence for making predictions. Furthermore, Bayesian optimization is employed to fine-tune hyperparameters, further improving forecast precision. Our results demonstrate a significant improvement in forecast precision over traditional recurrent neural networks. This indicates the potential of our integrated approach to effectively handle the complex patterns and dependencies in stock price data.

## 1. Introduction

The stock market is a complex and dynamic system influenced by numerous factors, making accurate prediction a challenging task. While traditional models have been widely used, their ability to capture the complex, non-linear, and time-varying dynamics of stock market behavior is often limited. The emergence of deep learning has opened new avenues for more sophisticated and accurate forecasting models [1]. Deep learning models have shown great promise in various domains, such as language modeling, machine translation, and image and speech recognition. An increasing number of studies have explored the application of deep learning techniques for stock price prediction [2,3,4]. These models leverage advanced architectures, such as Convolutional Neural Networks (CNN), Recurrent Neural Networks (RNN), Long Short-Term Memory (LSTM) networks, Gated Recurrent Units (GRU) and more recently, attention-based mechanisms, to better understand and predict stock market movements.

Recent advancements in sequence-to-sequence modeling, particularly the development of encoder–decoder architectures, have demonstrated remarkable success in various domains, including language modeling and machine translation. These architectures excel at capturing long-range dependencies within sequential data by effectively processing input sequences and generating corresponding output sequences. Inspired by these successes, this research investigates the applicability of encoder–decoder architectures, including those incorporating attention mechanisms, for predicting stock price indices in the Vietnamese market.

**This research addresses the following key research questions:**

1. Given their effectiveness in language modeling, such as predicting the next word in a sequence, can encoder–decoder architectures also excel in stock price prediction contexts?
2. Can encoder–decoder architectures, including those with and without attention mechanisms, outperform traditional recurrent neural networks in predicting stock price indices in the Vietnamese market?
3. What is the impact of attention mechanisms on the predictive performance of encoder–decoder architectures for stock price forecasting in the Vietnamese context?

**This research contributes to the existing literature in several key ways:**

- **Application to the Vietnamese market:** This study extends the application of advanced deep learning models, specifically encoder–decoder architectures with and without attention mechanisms, to the Vietnamese stock market, a market with unique characteristics and limited prior research on the application of these sophisticated models.
- **Comparative analysis:** We conduct a comprehensive comparative analysis of the performance of encoder–decoder models with and without attention mechanisms against traditional recurrent neural networks, providing valuable insights into the relative strengths and weaknesses of these different approaches.
- **Rigorous methodology:** We employ a rigorous experimental framework, including hyperparameter tuning using Bayesian optimization, to ensure optimal model performance and robust evaluation.

This research focuses on two major Vietnamese stock market indices: the VN-Index of the Ho Chi Minh Stock Exchange (HOSE) and the HNX-Index of the Hanoi Stock Exchange (HNX). These indices serve as crucial benchmarks for the Vietnamese stock market, providing a comprehensive overview of market performance. The findings of this study have the potential to inform investment decisions, enhance risk management strategies, and contribute to a deeper understanding of the dynamics of the Vietnamese stock market.

The rest of the paper is structured as follows: Section 2 reviews early deep learning models for stock price prediction, encoder–decoder architectures, and attention mechanisms, highlighting the research gap addressed by this paper. Section 3 discusses various types of recurrent neural networks, including RNN, LSTM, and GRU. Section 4 introduces the encoder–decoder architecture and attention mechanism. Section 5 describes the experimental setup, covering data processing, hyperparameter settings, and model performance measures. Section 6 presents the results. Finally, Section 7 concludes the paper.

## 2. Related Work

### 2.1. Significant Early Contributions to Deep Learning Models for Stock Price Prediction

Over the years, researchers have proposed various deep learning models to tackle the challenges of stock price prediction. Here, we review some of the most significant contributions that have laid the foundation for contemporary research in this field.

In 2020, Lu et al. [5] presented a novel forecasting model that combines CNN and LSTM networks to predict stock prices. The model leverages the CNN’s ability to extract spatial features from data and the LSTM’s capability to analyze temporal dependencies. The combined CNN-LSTM model effectively captures both spatial and temporal patterns in stock price data, leading to improved prediction accuracy compared to traditional models. In 2021, Lu et al. [6] introduced a refined method utilizing CNN, Bi-directional LSTM (BiLSTM), and an attention mechanism (AM) to enhance the prediction accuracy of stock prices. The integration of CNN for feature extraction, BiLSTM for capturing temporal dependencies, and attention mechanisms for focusing on crucial information significantly improved the model’s predictive performance.

In 2022, Wang et al. [7] proposed a deep learning model based on the Transformer architecture to predict stock market indices. The model leverages the self-attention mechanism of Transformers to capture complex patterns and dependencies in stock price data. The experimental results demonstrate that the Transformer-based model outperforms traditional methods and other deep learning models, such as RNN, LSTM and CNN, in terms of prediction accuracy. The study highlights the potential of Transformer models in financial time series forecasting. Kanwal et al. [8] introduced a hybrid deep learning model called BiCuDNNLSTM-1dCNN for predicting stock prices. The model combines a Bidirectional Cuda Deep Neural Network LSTM (BiCuDNNLSTM) with a one-dimensional CNN (1dCNN). This hybrid approach leverages the strengths of both architectures to improve the accuracy and efficiency of stock price predictions.

In 2023, Yang et al. [9] introduced a novel model integrating modern machine learning techniques to enhance the accuracy of stock price predictions. The model features a memory attention module to better capture long-term dependencies in time series data and a unique long-distance loss function to improve predictive precision. Wang et al. [10] introduced a novel prediction model called the Localized Graph Convolutional Network (LoGCN) for stock index forecasting. This model leverages a well-designed convolution mechanism to capture intricate local spatial-temporal connections, significantly improving the accuracy of stock market index predictions compared to traditional models.

In 2024, Zhu et al. [11] introduced PMANet, an advanced hybrid model designed for stock price prediction in the Chinese market. PMANet incorporates Multi-scale Timing Feature Attention, combining Multi-scale Timing Feature Convolution and Ant Particle Swarm Optimization to enhance the understanding of dependencies and interrelations within stock data sequences. The model also features a Probabilistic Positional Attention mechanism to better handle anomaly points in stock sequences. Chen et al. [12] proposed the CED-PSO-StockNet model to address the challenges of low prediction accuracy in noisy stock market environments. The model uses Complete Ensemble Empirical Mode Decomposition with Adaptive Noise (CEEMDAN) to decompose raw stock data and remove noise. The components are then reconstructed using the extreme point method to enhance stability. An encoder–decoder framework with an attention mechanism is employed to predict the reconstructed components accurately.

Xie et al. [13] introduced a novel model called the Deep Convolutional Transformer (DCT) for predicting stock movements. The DCT model combines CNN and transformer architectures with a multi-head attention mechanism. It features an inception convolutional token embedding architecture and separable fully connected layers. Results show that the DCT model achieves the highest accuracy and outperforms other models. Li and Xu [14] proposed a new approach to enhance stock price prediction by leveraging Generative Adversarial Networks (GANs) and transformer-based attention mechanisms. GANs are used to generate synthetic stock price data, incorporating market sentiment and volatility. The attention mechanisms selectively focus on important features and patterns in the data, aiding in the identification of key market indicators. By integrating market social media news, the proposed model outperforms conventional approaches.

### 2.2. Historical Developments in Encoder–Decoder Architecture and Attention Mechanism

The concept of attention mechanisms in neural networks has evolved significantly over time. Early attempts to incorporate attention into neural networks date back to the late 1980s, with studies such as the improved version of the Neocognitron with selective attention [15]. The encoder–decoder architecture itself was developed in the early 2010s, with pivotal contributions from papers published in 2014 that applied this architecture to sequence-to-sequence tasks like machine translation [16,17]. The integration of attention mechanisms into these architectures, as demonstrated by Bahdanau et al. [18], further enhanced their performance by enabling the model to focus on relevant parts of the input sequence during decoding. This method, known as the Bahdanau attention mechanism, significantly improved the basic encoder–decoder architecture by addressing the bottleneck of using a fixed-length vector to represent the entire input sequence.

Later, Cho et al. [19] introduced attention-based encoder–decoder networks for describing multimedia content. The authors focus on tasks such as machine translation, image caption generation, video clip description, and speech recognition. The model leverages GRUs and CNNs, along with trained attention mechanisms, to learn to attend to different parts of the input for each element of the output. However, the modern attention-based encoder–decoder framework gained prominence with the introduction of the Transformer model in the 2017 paper “Attention is All You Need” by Vaswani et al. [20], which laid the foundation for many subsequent advancements in attention-based models. The success of these models in handling sequential data inspired researchers to explore their potential in time series prediction [21,22,23].

In 2020, Du et al. [24] proposed a novel attention-based encoder–decoder framework for multivariate time series forecasting. The model integrates traditional encode context vectors with temporal attention vectors for joint temporal representation learning. It uses BiLSTM layers with a temporal attention mechanism as the encoder network to adaptively learn long-term dependencies and hidden correlation features of multivariate temporal data. The model was tested on five typical multivariate time series datasets and showed superior forecasting performance compared to baseline methods.

In 2021, Jin et al. [25] proposed a novel deep-learning model for short-term electric power load forecasting. The model uses an attention-based encoder–decoder architecture with GRU to capture temporal dependencies in time-series data. A temporal attention layer is employed to focus on key features, enhancing prediction accuracy. Bayesian optimization is used to fine-tune the model’s hyperparameters, ensuring optimal performance. The model was tested on real power load data from American Electric Power (AEP) and demonstrated superior accuracy and stability compared to existing methods.

In 2023, Wu and Zhang [26] proposed a new model using attention-based encoder–decoder networks to estimate the state of charge (SOC) of lithium-ion batteries under complex ambient temperature conditions. The model employs a BiLSTM encoder to obtain the hidden state vector from an input sequence. The attention mechanism helps in focusing on relevant features, improving the accuracy of SOC estimation. Klaar et al. [27] proposed an optimized model for predicting faults in insulators using Empirical Wavelet Transform (EWT), Sequence-to-Sequence (Seq2Seq) architecture, LSTM networks, and an attention mechanism. The model aims to reduce the influence of non-representative variations in the data and improve prediction accuracy. The Optuna framework is used for hyperparameter optimization, resulting in a significant reduction in MSE compared to standard LSTM models and models without optimization.

In 2024, Jayanth and Manimaran [28] introduced a hybrid model that combines Double Exponential Smoothing (DES) with a deep learning model called Dual Attention encoder–decoder-based Bi-directional Gated Recurrent Unit (DA-ED-Bi-GRU), optimized using Bayesian Optimization. The model aims to efficiently identify patterns and trends in stock data. The DES method handles trends and seasonality, while the DA-ED-Bi-GRU captures intricate patterns in the stock data. The parameters are optimized using Bayesian optimization to maximize the model’s performance. The proposed hybrid model was tested on stock price data from General Electric, Microsoft, and Amazon, showing reasonable accuracy in predictions.

There are limited existing studies that integrate the encoder, decoder, and attention mechanism for stock market forecasting, with none focusing on the Vietnamese stock market. Our research addresses this gap by adapting this innovative architecture into novel models to predict the next trading day’s stock price index, which is distinct from previous studies.

## 3. Recurrent Neural Networks

Stock market index forecasting poses several challenges that require advanced modeling techniques. One key challenge is capturing the temporal dependencies inherent in stock price data, which fluctuates over time and can exhibit complex patterns. Additionally, managing the noise present in these data is crucial, as it can obscure the underlying trends and lead to inaccurate predictions.

To address these challenges, researchers often employ RNNs and their variants, such as LSTM networks and GRUs. These models are particularly well-suited for capturing temporal dependencies due to their ability to maintain and update hidden states over time. They can also implicitly learn to filter out noise in the input data. By training on noisy stock price data, the network can identify and de-emphasize irrelevant fluctuations, focusing on the underlying trends and patterns. Leveraging these advanced techniques enables more accurate and robust forecasts of stock market indices, effectively addressing the specific challenges of temporal dependency and data noise.

### 3.1. RNN

**Figure 1:**
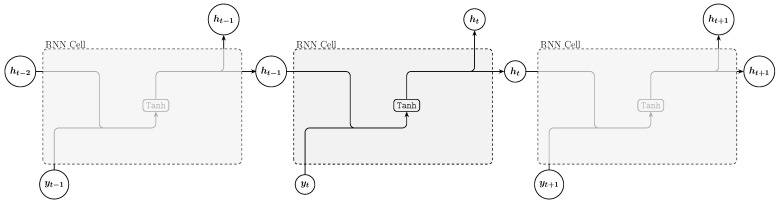
RNN architecture.

A Recurrent Neural Network (RNN) is a fundamental type of neural network designed for processing sequential data, making it suitable for tasks such as language modeling, machine translation, speech recognition, and time series prediction. Its core feature is the hidden state, given by Equation (1), which captures information from previous inputs to influence the current output, essentially giving the network a form of memory (Figure 1).

(1) $$\begin{array}{cc} h_{t} & =\sigma \left(W_{h}\cdot \left[h_{t-1},y_{t}\right]+b_{h}\right) \end{array}$$

where σ is the activation function (commonly tanh or ReLU), $W_{h}$ is the weight matrix, $b_{h}$ is the bias term, $h_{t-1}$ is the previous hidden state, and $y_{t}$ is the current input.

**Figure 2:**
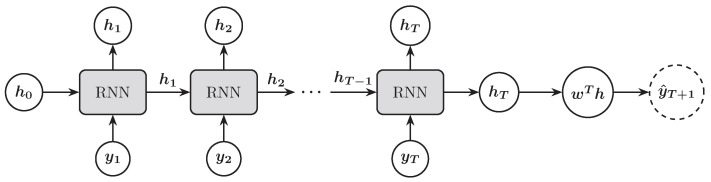
Illustration of an RNN architecture for forecasting the next trading day’s stock price index.

Figure 2 illustrates the process of using the RNN architecture to predict the next trading day’s stock price index. The RNN transforms the input sequence $\left\{y_{1},y_{2},\ldots ,y_{T}\right\}$ into a final hidden state ($h_{T}$) that represents the information of the entire input sequence. The final hidden state is then passed through an affine transformation to produce the forecast value for the next trading day ${\hat{y}}_{T+1}$.

### 3.2. LSTM

**Figure 3:**
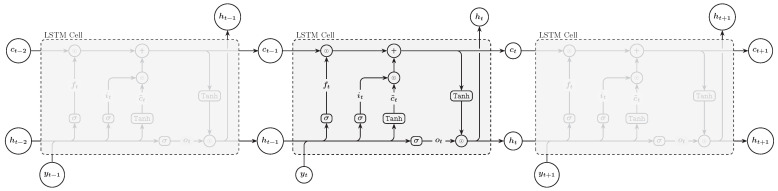
LSTM architecture.

Long Short-Term Memory (LSTM) network, introduced by Hochreiter and Schmidhuber in 1997, is a type of recurrent neural network capable of learning order dependence in sequence prediction problems. Unlike a standard RNN that struggles with long-term dependencies due to the vanishing gradient problem, LSTM has a unique architecture that allows it to remember information for longer periods. This architecture is composed of three gates: the forget gate, which determines what information is discarded from the cell state; the input gate, which controls the flow of new information into the cell; and the output gate, which decides what information is going to be output based on the cell state and the input [29]. The update steps of LSTM can be expressed as follows (Figure 3):

(2) $$\begin{array}{cc} f_{t} & =\sigma \left(W_{f}\cdot \left[h_{t-1},y_{t}\right]+b_{f}\right) \end{array}$$

(3) $$\begin{array}{cc} i_{t} & =\sigma \left(W_{i}\cdot \left[h_{t-1},y_{t}\right]+b_{i}\right) \end{array}$$

(4) $$\begin{array}{cc} o_{t} & =\sigma \left(W_{o}\cdot \left[h_{t-1},y_{t}\right]+b_{o}\right) \end{array}$$

(5) $$\begin{array}{cc} {\tilde{c}}_{t} & =\tanh\left(W_{c}\cdot \left[h_{t-1},y_{t}\right]+b_{c}\right) \end{array}$$

(6) $$\begin{array}{cc} c_{t} & =f_{t}⊙c_{t-1}+i_{t}⊙{\tilde{c}}_{t} \end{array}$$

(7) $$\begin{array}{cc} h_{t} & =o_{t}⊙\tanh\left(c_{t}\right) \end{array}$$

where σ is the sigmoid function, *W* and *b* are weights and biases, respectively, $h_{t-1}$ is the previous hidden state, and $y_{t}$ is the current input, ⊙ denotes the Hadamard product (element-wise product).

**Figure 4:**
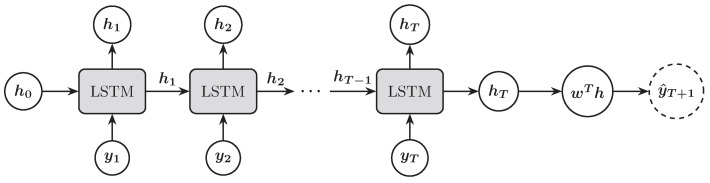
Illustration of an LSTM architecture for forecasting the next trading day’s stock price index.

Given an input sequence $\left\{y_{1},y_{2},\ldots ,y_{T}\right\}$, the LSTM transforms it into a final hidden state ($h_{T}$) that captures the information of the entire sequence. This final hidden state is then passed through an affine transformation to generate the forecast value for the next trading day’s stock price index (Figure 4).

### 3.3. Gated Recurrent Units

**Figure 5:**
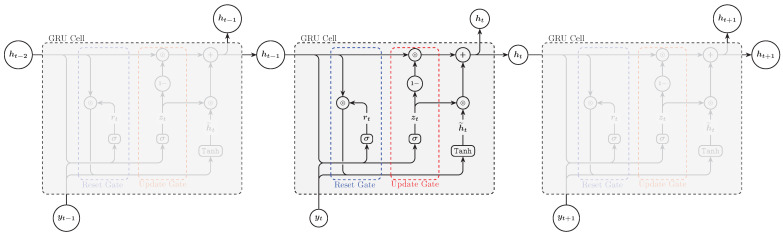
GRU architecture.

Gated Recurrent Unit (GRU) is another type of RNN that was introduced by Cho et al. in 2014. GRU is simpler than LSTM but still effective at capturing long-term dependencies in sequences. Unlike the LSTM, which utilizes three gates (input, forget, and output), GRU simplifies the architecture by employing only two gates: the reset gate, which determines how much of the previous hidden state to forget and the update gate, which controls how much of the previous hidden state and the new candidate hidden state to use (Figure 5). This streamlined design not only reduces the computational complexity but also accelerates the training process while maintaining comparable performance to LSTM [30]. The update steps of GRU can be expressed as follows:

(8) $$\begin{array}{cc} r_{t} & =\sigma \left(W_{r}\cdot \left[h_{t-1},y_{t}\right]+b_{r}\right) \end{array}$$

(9) $$\begin{array}{cc} z_{t} & =\sigma \left(W_{z}\cdot \left[h_{t-1},y_{t}\right]+b_{z}\right) \end{array}$$

(10) $$\begin{array}{cc} {\tilde{h}}_{t} & =\tanh\left(W_{h}\left[r_{t}⊙h_{t-1},y_{t}\right]+b_{h}\right) \end{array}$$

(11) $$\begin{array}{cc} h_{t} & =\left(1-z_{t}\right)⊙h_{t-1}+z_{t}⊙{\tilde{h}}_{t} \end{array}$$

where σ is the sigmoid function, *W* and *b* are weights and biases, respectively, $h_{t-1}$ is the previous hidden state, and $y_{t}$ is the current input.

**Figure 6:**
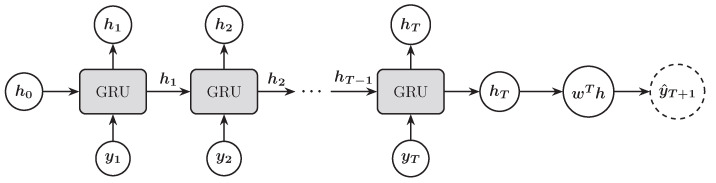
Illustration of a GRU architecture for forecasting the next trading day’s stock price index.

The efficacy of GRU has been demonstrated across various applications, including natural language processing, time series forecasting, and speech recognition. Their ability to efficiently manage long-term dependencies makes them a valuable tool in the arsenal of deep learning techniques. Figure 6 illustrates the process of using the GRU architecture to predict the next trading day’s stock price index. The process follows the same methodology as described for the RNN and LSTM architectures.

## 4. Encoder–Decoder Architecture

**Figure 7:**
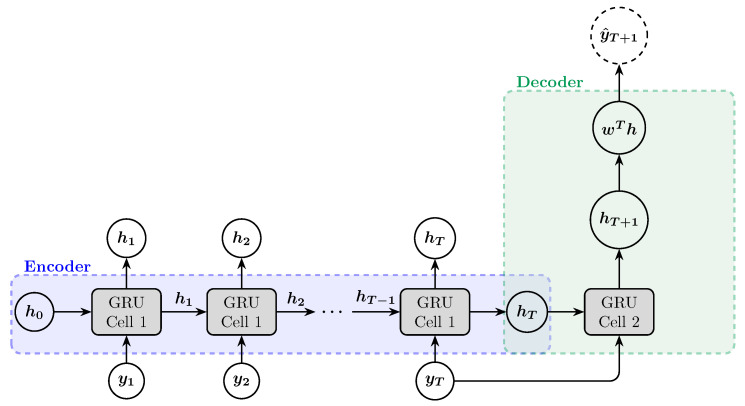
Encoder–decoder architecture for forecasting the next trading day’s stock price index. In this architecture, the encoder’s final hidden state is used as the initial hidden state for the decoder, and the decoder’s input value is the last value of the input sequence.

The encoder–decoder architecture is a fundamental framework in deep learning, particularly effective for sequence-to-sequence tasks such as machine translation, text summarization, and image captioning. This architecture consists of two main components: the encoder and the decoder, each typically implemented using RNN, LSTM, or GRU. Figure 7 illustrates the flow of information from the encoder to the decoder.

### 4.1. Encoder

The encoder processes the input sequence and compresses it into a final hidden state ($h_{T}$), which encapsulates the essential information of the input sequence. Formally, given an input sequence $\left\{y_{1},y_{2},\ldots ,y_{T}\right\}$, the encoder generates a sequence of hidden states $\left\{h_{1},h_{2},\ldots ,h_{T}\right\}$ using the following recurrence relation:

(12) $$h_{t}=f\left(y_{t},h_{t-1}\right),\;$$

where $h_{t}$ is the hidden state at time step *t*, $y_{t}$ is the input at time step *t*, and *f* is a non-linear function such as an RNN, LSTM or GRU cell, and in this study, it is the GRU cell (Figure 7).

### 4.2. Decoder

For a one-step forecast, the decoder generates the output (${\hat{y}}_{T+1}$) based on the final hidden state ($h_{T}$) of the encoder and the last input ($y_{T}$):

(13) $$\begin{array}{cc} h_{T+1} & =f(y_{T},h_{T}) \end{array}$$

(14) $$\begin{array}{cc} {\hat{y}}_{T+1} & =g(h_{T+1}) \end{array}$$

where *f* is a non-linear function such as an RNN, LSTM or GRU cell. In this study, it is also the GRU cell, and *g* is an affine transformation (Figure 7).

### 4.3. Attention

The encoder–decoder architecture is quite fascinating, but it faces a limitation (a bottleneck): the entire input sequence is compressed into one hidden state, the encoder’s final hidden state. Consequently, the decoder produces the output with minimal information. To enhance the performance of the encoder–decoder architecture, an attention mechanism can be incorporated. This mechanism allows the decoder to focus on different parts of the encoder’s hidden state at the decoding step, rather than relying solely on the final hidden state of the encoder. The attention mechanism computes a context vector ($c_{T+1}$) for the output of the decoder as follows: Each encoder’s hidden state ($h_{i}$) is transformed into a key ($K_{i}$) using an affine transformation and into a value ($V_{i}$) using an identity transformation, and the decoder’s hidden state ($h_{T+1}$) is transformed into a query ($Q_{T+1}$) using another affine transformation. Then, the alignment score ($s_{i}$) that measures the relevance of the encoder hidden state $h_{i}$ to the decoder’s state $h_{T+1}$ is computed as the dot product of the query vector and the key vector:

(15) $$s_{i}=Q_{T+1}\cdot K_{i}$$

The dot product is then scaled by the square root of the dimension of the key vectors ($d_{k}$) to prevent the gradients from becoming too small during backpropagation:

(16) $$s_{i}=\frac{Q_{T+1}\cdot K_{i}}{\sqrt{d_{k}}}$$

The attention scores ($\alpha_{i}$) are calculated by using the softmax function:

(17) $$\alpha_{i}=\frac{\exp(s_{i})}{\sum_{k=1}^{T}\exp\left(s_{k}\right)}$$

Finally, the context vector is calculated as a weighted sum of the values (the encoder’s hidden states):

(18) $$c_{T+1}=\sum_{i=1}^{T}\alpha_{i}V_{i}$$

**Figure 8:**
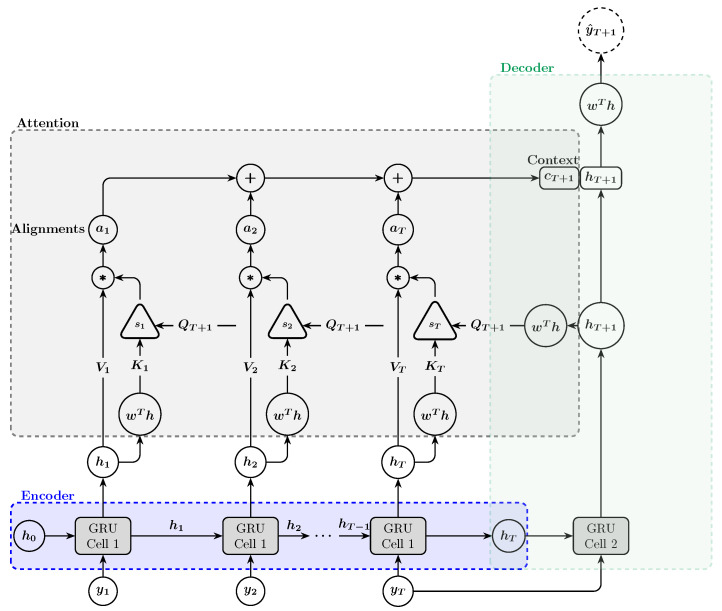
Encoder–decoder architecture with a single-head attention mechanism for forecasting the next trading day’s stock price index. In this architecture, the encoder’s final hidden state is used as the initial hidden state for the decoder, and the decoder’s input value is the last value of the input sequence.

The context vector ($c_{T+1}$) is concatenated with the decoder’s hidden state ($h_{T+1}$) and then passed through an affine transformation to generate the forecast value (${\hat{y}}_{T+1}$), as illustrated in Figure 8.

For stock price forecasting, the encoder reads and compresses the input sequence (historical stock prices) into a fixed-length hidden vector. The decoder then generates the output (future stock price) from this vector. This architecture is beneficial for handling variable-length input and output sequences, capturing dependencies across different time scales, and being flexible in addressing complex forecasting tasks. Attention mechanisms enhance the encoder–decoder architecture by allowing the model to focus on the most relevant parts of the input sequence when making predictions. This is particularly beneficial for stock price forecasting, where certain past data points may be more indicative of future trends than others. By dynamically weighing the importance of these data points, the attention mechanism improves the model’s ability to capture intricate patterns and dependencies, leading to potentially more accurate forecasts.

## 5. Experiments

We conduct an experiment to study the performance of the proposed models and compare them with the RNN, LSTM and GRU models. Table 1 details the hardware and software configurations used for the stock price prediction experiments to ensure reproducibility and to highlight the computational environment.

### 5.1. Datasets

**Figure 9:**
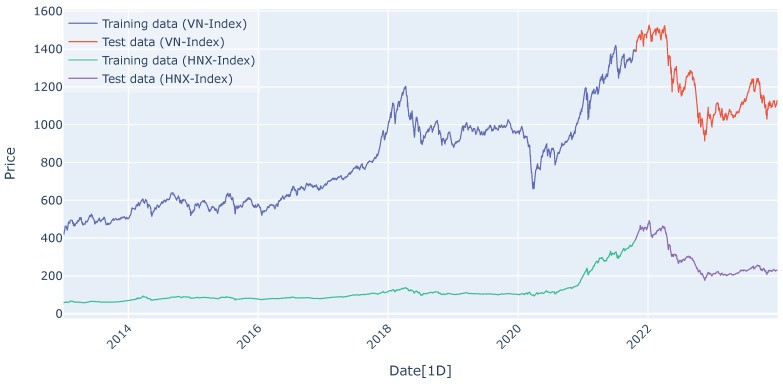
Time series plots for VN-Index and HNX-Index data.

In this study, we use two main Vietnamese stock market indices, the VN-Index and HNX-Index, to demonstrate the performance of integrating the encoder, the decoder, and the attention mechanism. We collected the daily closing prices of both indices over the period from 1 January 2013, to 31 December 2023. We retrieved the data at the start of 2024, making 2023 the ending point to ensure we had the most recent full year of data available. We selected 2013 as the starting point due to the limitations of our computational resources for training the models. This period covers a decade, allowing us to capture and analyze long-term trends and significant market shifts, providing a comprehensive analysis for our research. The summaries of these datasets are provided in Table 2. Figure 9 displays the time series plots for the VN-Index and HNX-Index data, highlighting the training and test sets.

### 5.2. Data Preprocessing

Each dataset is divided into two sets: the first 80% of the data is used for training, while the remaining 20% is used for testing. The training and test sets are standardized to have zero mean and unit variance. This process improves the stability and efficiency of training, enhances the performance of optimization algorithms, helps prevent overfitting, and leads to better model performance and generalization. The standard score of a sample *y* is calculated as:

(19) $$z=\frac{y-\bar{y}}{s}$$

where $\bar{y}$ and *s* are the mean and standard deviation of the training set respectively. After making predictions, it is crucial to inverse-transform the predicted values back to their original scale. This step ensures that the predicted values can be interpreted in the context of the original data. To reverse the standardization and retrieve the original scale, we use the formula:

(20) $$y=z\cdot s+\bar{y}$$

**Figure 10:**
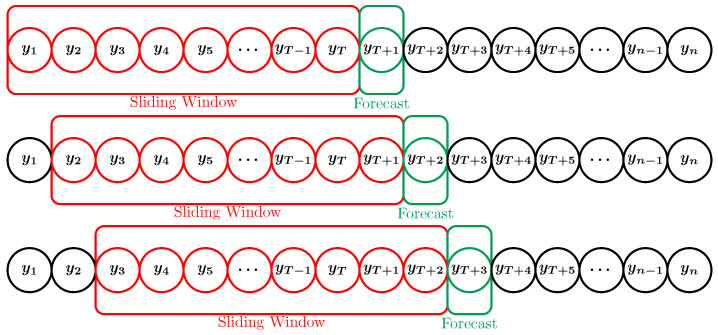
A sliding window is used to generate the input and target output from the observed time series.

Suppose $\left\{y_{1},y_{2},\ldots ,y_{n}\right\}$ represents the standardized data of the training set or test set. We use a sliding window approach to generate the input and target output from this data, thereby transforming the time series data into a supervised learning problem, as illustrated in Figure 10. In this study, we utilize a sliding window approach with a window size of 5, meaning each prediction is based on the previous 5 time steps. Our pilot study determined this window size by observing the test loss for various sizes until there was negligible improvement. This choice helps capture local trends and temporal dependencies within the data, enabling the model to learn from recent price history, and facilitates pair comparison between models. According to [31], larger look-back periods may lead to poor stock price prediction performance, wasting valuable training and prediction time.

### 5.3. Hyperparameters Setting

For the VN-Index, it was straightforward to determine the optimal hyperparameters by observing the test loss curve through trial and error. However, this approach was not effective for the HNX-Index, as the patterns in the training and test sets differed significantly, as shown in Figure 9. Consequently, the optimal hyperparameters for the HNX-Index were identified using Bayesian optimization, a probabilistic model that iteratively refines its estimates to find the best set of parameters. Detailed information on this method can be found in [25,28]. Table 3 details the hyperparameters used in RNN, LSTM, GRU, encoder–decoder, and encoder–decoder attention architectures for the VN-Index and HNX-Index predictions. Hyperparameters include hidden layers, hidden size, batch size, loss function, optimizer type, learning rate, number of epochs, period for learning rate decay, and the multiplicative factor of learning rate decay. Due to the limitation of computational resources, we select a single hidden layer, a batch size of 32, MSE loss, and the Adam optimizer.

In Table 3, we employ Bayesian optimization to fine-tune the hidden size, learning rate, learning rate decay period, and the multiplicative factor of learning rate decay. The parameter search space is defined as follows: hidden size (1–300), learning rate (0.00001–0.1), learning rate decay period (1–400), and its multiplicative factor (0.1–1). The objective function aims to minimize the test loss. We set the stopping criteria based on a fixed number of iterations, incrementally increasing this number until the test loss shows no improvement. This comprehensive approach ensures optimal hyperparameters, enhancing the model’s performance and robustness.

Choosing an optimal learning rate is quite challenging. On the one hand, a learning rate that is too high can cause the optimizer to overshoot and miss the optimal point, resulting in erratic behavior and a longer time to converge. On the other hand, a learning rate that is too low can cause the optimizer to get stuck in a suboptimal solution. Therefore, we set a scheduler to decrease the learning rate by defining the period of learning rate decay and its multiplicative factor to ensure that all loss functions converge faster and reach the optimal solution. The period of learning rate decay and its multiplicative factor are also hyperparameters. That is why we include them in Bayesian optimization.

### 5.4. Model Performance Measures

In the literature, mean absolute error (MAE), root mean square error (RMSE), and mean absolute percentage error (MAPE) are commonly used metrics for evaluating the performance of forecasting models. Their calculations are shown as follows:

(21) $$\begin{array}{cc} \; & \mathrm{MAE}=\frac{1}{n}\sum_{i=1}^{n}\left|y_{i}-{\hat{y}}_{i}\right| \end{array}$$

(22) $$\begin{array}{cc} \; & \mathrm{RMSE}=\sqrt{\frac{1}{n}\sum_{i=1}^{n}{\left(y_{i}-{\hat{y}}_{i}\right)}^{2}} \end{array}$$

(23) $$\begin{array}{cc} \; & \mathrm{MAPE}=\frac{1}{n}\sum_{i=1}^{n}\left|\frac{y_{i}-{\hat{y}}_{i}}{y_{i}}\right|\times 100 \end{array}$$

where ${\hat{y}}_{i}$ is the predicted value, and $y_{i}$ is the actual value. MAE provides a straightforward measure of average error magnitude, making it easy to interpret. RMSE emphasizes larger errors more due to the squaring of differences, which can be particularly useful when larger errors are highly undesirable. MAPE gives a percentage error, offering a relative measure of error that can be easier to interpret across different scales. Smaller values of MAE, RMSE, and MAPE indicate better forecasting performance.

## 6. Results

**Figure 11:**
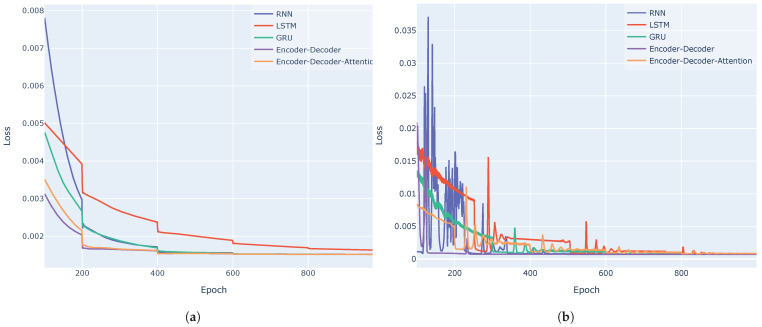
Training loss curves of the five models for VN-Index (**a**) and HNX-Index (**b**) using schedulers to decrease the learning rate.

Figure 11 shows the training loss curves for the VN-Index (a) and the HNX-Index (b) when applying schedulers to decrease the learning rates. It is observed that with the scheduler, the training loss functions converge faster within 1000 epochs. Table 4 presents the means and standard errors of three metrics evaluated across 10 experiments on the test sets of two datasets. This table illustrates the consistency and reliability of the models’ performance across different runs. It is evident that the encoder–decoder architectures, both with and without the attention mechanism, generally perform better across both datasets, achieving lower MAE, RMSE, and MAPE values compared to the RNN, LSTM, and GRU models.

**Figure 12:**
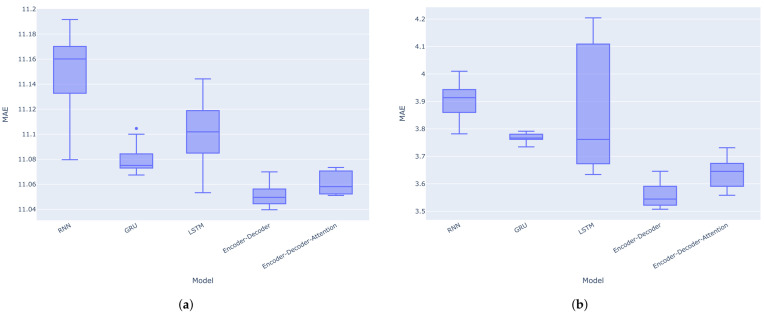
Boxplots for the MAE of the models across 10 separate experiments on the VN-Index (**a**) and HNX-Index (**b**) test sets.

**Figure 13:**
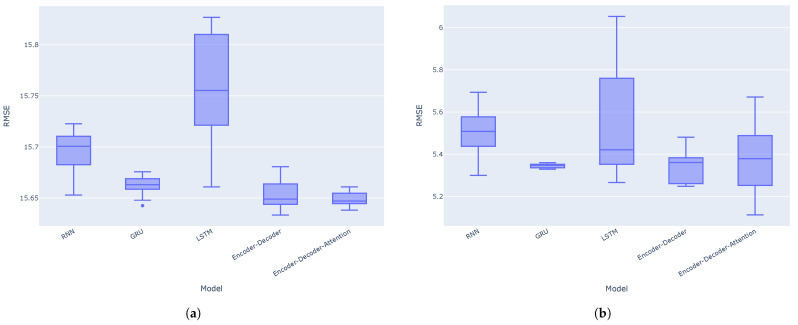
Boxplots for the RMSE of the models across 10 separate experiments on the VN-Index (**a**) and HNX-Index (**b**) test sets.

**Figure 14:**
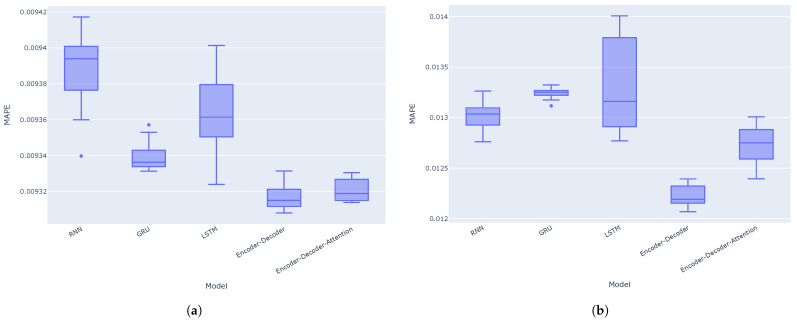
Boxplots for the MAPE of the models across 10 separate experiments on the VN-Index (**a**) and HNX-Index (**b**) test sets.

The results indicate that the encoder–decoder architecture without the attention mechanism performs even better than with the attention mechanism for these one-step predictions. This could be due to the nature of one-step forecasting. The encoder’s final hidden state may already provide sufficient information for the decoder to generate the next forecast value. Therefore, adding an attention mechanism to the architecture might not yield an improvement, and could potentially even degrade performance. Attention mechanisms may be more appropriate for multi-step forecasting or seasonal data. Moreover, the GRU model consistently outperforms the RNN and LSTM models across all metrics (MAE, RMSE, and MAPE) for both the VN-Index and HNX-Index. Boxplots for the MAE, RMSE, and MAPE calculated across 10 separate experiments on the VN-Index (a) and HNX-Index (b) test sets are shown in Figure 12, Figure 13 and Figure 14, respectively. It is again observed that the encoder–decoder architectures, both with and without the attention mechanism, outperform the traditional recurrent neural networks. The LSTM model shows the highest variance in terms of MAE, RMSE, and MAPE among the models in most cases.

**Figure 15:**
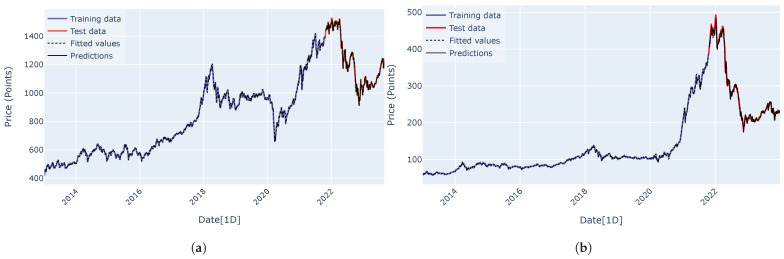
The predicted curves, along with the training and test data plots for the VN-Index (**a**) and HNX-Index (**b**), were generated using the RNN architecture.

**Figure 16:**
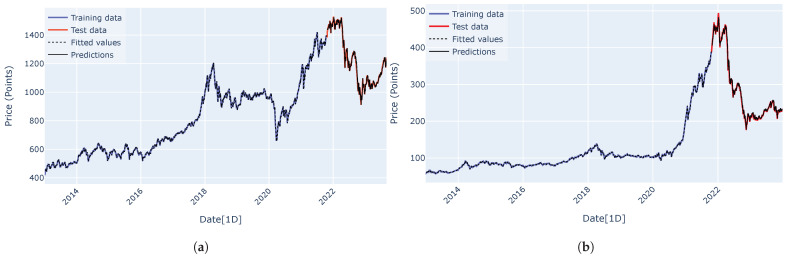
The predicted curves, along with the training and test data plots for the VN-Index (**a**) and HNX-Index (**b**), were generated using the LSTM architecture.

**Figure 17:**
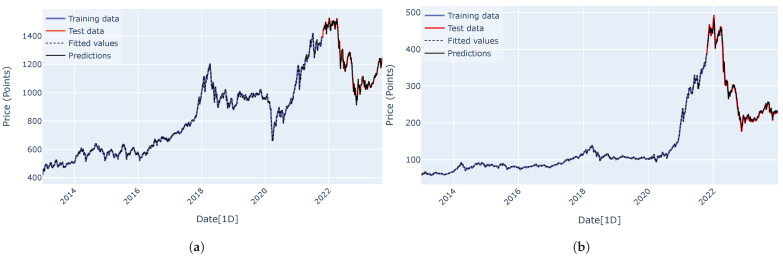
The predicted curves, along with the training and test data plots for the VN-Index (**a**) and HNX-Index (**b**), were generated using the GRU architecture.

**Figure 18:**
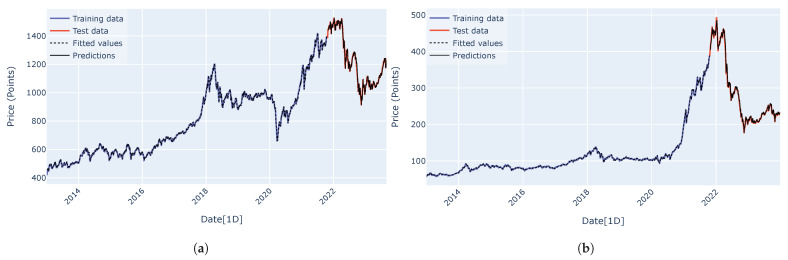
The predicted curves, along with the training and test data plots for the VN-Index (**a**) and HNX-Index (**b**), were generated using the encoder–decoder architecture.

**Figure 19:**
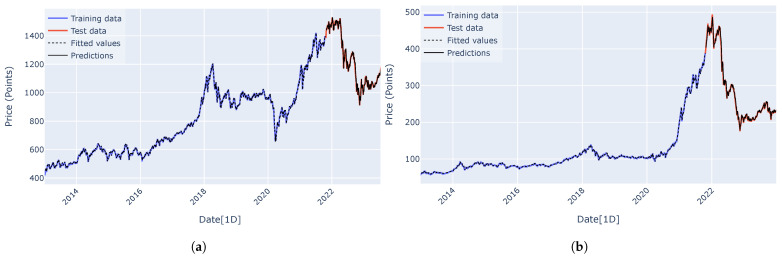
The predicted curves, along with the training and test data plots for the VN-Index (**a**) and HNX-Index (**b**), were generated using the encoder–decoder architecture with an attention mechanism.

**Figure 20:**
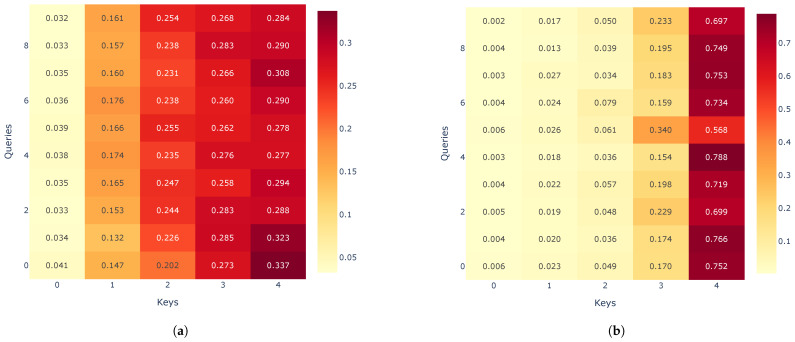
The attention score matrices for the first ten predictions on the test set, made by the encoder–decoder architecture with an attention mechanism, are shown for both the VN-Index (**a**) and the HNX-Index (**b**). For each row, the attention scores indicate how the decoder’s hidden state focuses on each of the five hidden states of the encoder.

Predicted curves for the VN-Index (a) and the HNX-Index (b) using the RNN, LSTM, GRU, encoder–decoder architecture, and encoder–decoder architecture with attention mechanism are shown in Figure 15, Figure 16, Figure 17, Figure 18 and Figure 19. It is observed that all the models fit well to the datasets, and the encoder–decoder model, both with and without the attention mechanism, demonstrates the best performance. Figure 20 shows the attention score matrices for the first ten predictions on the test set made by the encoder–decoder architecture with the attention mechanism, for both the VN-Index (a) and the HNX-Index (b). For each row, the attention scores indicate how the decoder’s hidden state attends to each of the five hidden states of the encoder. The attention score matrices reveal that for both the VN-Index and HNX-Index test sets, the model primarily focuses on the most recent observations to make predictions. This is particularly evident in cases where stock prices fluctuate sharply. This behavior is consistent across both datasets, emphasizing the model’s reliance on the latest data points. Consequently, we do not need an extensive history of inputs to forecast the next trading day’s stock price.

## 7. Conclusions

This study details the architecture of integrating the encoder, decoder and attention mechanism, discusses its theoretical underpinnings, and demonstrates its effectiveness for Vietnamese stock price prediction. Our experimental results indicate that this approach significantly enhances forecasting accuracy compared to traditional models such as RNN, LSTM, and GRU. These findings underscore the potential of advanced deep learning techniques in financial forecasting. A notable advantage of our approach is the use of Bayesian optimization, which efficiently determines the optimal set of hyperparameters, significantly contributing to the model’s performance. However, due to limitations in computational resources, we were unable to include all hyperparameters into the Bayesian optimization process.

By delivering a more accurate forecasting model, this study provides valuable insights for investors, financial analysts, and policymakers, enabling better-informed decisions regarding future financial trends and strategies. However, our findings are limited to the Vietnamese stock market. Future work is needed to evaluate the generalizability of this approach to other financial markets and to incorporate additional data sources, such as social media sentiment and financial news.

Furthermore, the results of the encoder–decoder architectures with and without the attention mechanism are inconsistent. Adding an attention mechanism to the architecture does not provide a significant improvement in one-step forecasting of the stock price index. Further research can be conducted to investigate the factors that influence the effectiveness of attention by conducting more extensive hyperparameter tuning, experimenting with different attention mechanisms (e.g., location-based attention, multi-head attention), and exploring multi-step forecasting.

## Figures and Tables

**Table 1:** Hardware and software configurations for experiments.

Component	Specification
CPU	AMD Ryzen 7 7435HS (3.10 GHz up to 4.50 GHz, 8 cores)
GPU	NVIDIA GeForce RTX 4060 (8 GB GDDR6)
Miniconda Version	Conda 24.9.2
Python Version	Python 3.12.7
PyTorch Version	PyTorch 2.5.1
Operating System	Window 11
CUDA Version	CUDA 11.6
Other Libraries	NumPy 1.26.4, Pandas 2.2.2, scikit-learn 1.5.1, scikit-optimize 0.10.2

**Table 2:** Descriptive statistics.

Index	Mean	Std	Min	$Q_{1}$	$Q_{2}$	$Q_{3}$	Max
VN-Index	873.0339	283.2851	418.3500	596.8300	899.9200	1064.0300	1528.5700
HNX-Index	146.9450	98.5540	57.6100	82.8200	104.5150	207.4400	493.8400

**Table 3:** Hyperparameter setting.

Model	Hyper-Parameter	VN-Index	HNX-Index
RNN	Hidden layers	1	1
	Hidden size	128	185
	Batch size	32	32
	Loss function	MSE	MSE
	Optimizer	Adam	Adam
	Learning rate	0.0001	0.0005
	Number of epochs	1000	700
	Period of learning rate decay	200	113
	Multiplicative factor of learning rate decay	0.5	0.33
LSTM	Hidden layers	1	1
	Hidden size	128	48
	Batch size	32	32
	Loss function	MSE	MSE
	Optimizer	Adam	Adam
	Learning rate	0.0001	0.0005
	Number of epochs	1500	2000
	Period of learning rate decay	200	253
	Multiplicative factor of learning rate decay	0.6	0.5
GRU	Hidden layers	1	1
	Hidden size	128	184
	Batch size	32	32
	Loss function	MSE	MSE
	Optimizer	Adam	Adam
	Learning rate	0.0001	0.0002
	Number of epochs	1000	1600
	Period of learning rate decay	200	300
	Multiplicative factor of learning rate decay	0.5	0.43
Encoder-Decoder	Hidden layers	1	1
	Hidden size	128	191
	Batch size	32	32
	Loss function	MSE	MSE
	Optimizer	Adam	Adam
	Learning rate	0.0001	0.00039
	Number of epochs	402	1000
	Period of learning rate decay	200	125
	Multiplicative factor of learning rate decay	0.5	0.145
Encoder-Decoder-Attention	Hidden layers	1	1
	Hidden size	128	128
	Batch size	32	32
	Loss function	MSE	MSE
	Optimizer	Adam	Adam
	Learning rate	0.0001	0.0001
	Number of epochs	402	2400
	Period of learning rate decay	200	200
	Multiplicative factor of learning rate decay	0.5	0.6

**Table 4:** The means and standard errors of three metrics (MAE, RMSE and MAPE) were evaluated across 10 separate experiments on the test sets of both datasets.

Dataset	Model	MAE	RMSE	MAPE
VN-Index	RNN	11.1491 (0.0319)	15.6967 (0.0209)	0.0094 (0.0000)
	LSTM	11.1006 (0.0268)	15.7524 (0.0518)	0.0094 (0.0000)
	GRU	11.0749 (0.0129)	15.6599 (0.0164)	0.0093 (0.0000)
	Encoder-Decoder	11.0512 (0.0091)	15.6529 (0.0132)	0.0093 (0.0000)
	Encoder-Decoder-Attention	11.0602 (0.0083)	15.6494 (0.0071)	0.0093 (0.0000)
HNX-Index	RNN	3.8963 (0.0652)	5.4988 (0.1112)	0.0130 (0.0001)
	LSTM	3.8539 (0.2091)	5.5601 (0.2694)	0.0133 (0.0005)
	GRU	3.7678 (0.0148)	5.3455 (0.0095)	0.0132 (0.0001)
	Encoder-Decoder	3.5612 (0.0478)	5.3473 (0.0725)	0.0122 (0.0001)
	Encoder-Decoder-Attention	3.6273 (0.0395)	5.3463 (0.1265)	0.0127 (0.0001)

## Data Availability

The raw data supporting the conclusions of this article will be made available by the authors upon request.

## Abbreviations

The following abbreviations are used in this manuscript:

RNN	Recurrent Neural Network
LSTM	Long Short-Term Memory
GRU	Gated Recurrent Unit
MAE	Mean Absolute Error
MSE	Mean Square Error
RMSE	Root Mean Square Error
MAPE	Mean Absolute Percentage Error
VN-Index	Ho Chi Minh Stock Exchange Index
HNX-Index	Hanoi Stock Exchange Index
